# Bone morphogenetic protein 8B (BMP8B) increases the glucose sensitivity of ventromedial hypothalamus (VMH) glucose-inhibited (GI) neurons in female mice.

**DOI:** 10.17912/micropub.biology.001496

**Published:** 2025-03-18

**Authors:** Pamela R Hirschberg, Munir Rahbe, Kevin Knapp, Hamad Wajid, Vanessa H Routh, Pallabi Sarkar

**Affiliations:** 1 Pharmacology, Physiology and Neuroscience, Rutgers Health, Newark, New Jersey, United States

## Abstract

In female, but not male, mice, bone morphogenetic protein (BMP) 8B affects energy homeostasis by inhibiting AMP activated protein kinase (AMPK) in the ventromedial hypothalamus (VMH). VMH glucose-inhibited (GI) neurons that express neuronal nitric oxide synthase (nNOS) increase blood glucose in an AMPK dependent fashion. We tested the hypothesis that BMP8B increases glucose inhibition on VMH nNOS-GI neurons. We found that more VMH nNOS neurons expressed the BMP8B receptor in females than males. Moreover, BMP8B blunted activation of VMH GI neurons in low glucose. Thus, VMH nNOS-GI neurons may mediate some of the metabolic effects of BMP8B in females.

**Figure 1. BMP8B increases the glucose sensitivity of vlVMH GI neurons:**
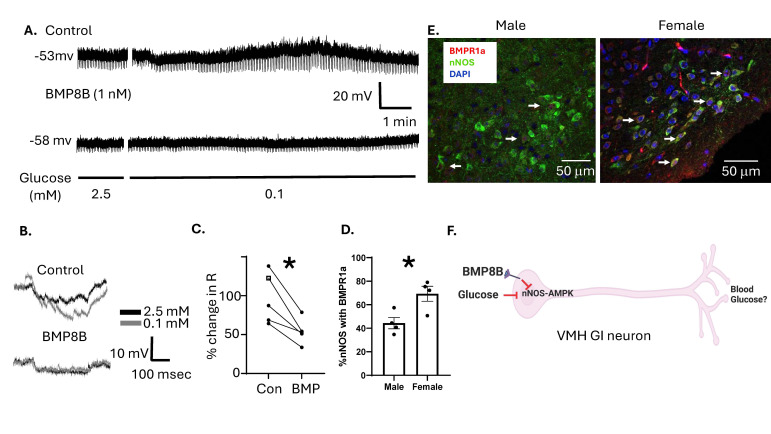
A. Representative whole cell current clamps traces from a glucose inhibited (GI) neuron in the ventrolateral ventromedial hypothalamus (vlVMH) in response to a glucose decrease from 2.5 to 0.1 mM glucose in the presence and absence of 1 nM bone morphogenetic protein (BMP) 8B. Top trace: This neuron depolarized and increased resistance in 0.1 mM glucose but returned to baseline before glucose was returned to 2.5 mM indicating that it belongs to the subcategory of adapting (Ad) GI neurons. Bottom trace: This depolarization and increased resistance was blocked in the presence of BMP8B. B. Representative voltage responses to a constant -20 pA hyperpolarizing current pulse in 2.5- and 0.1-mM glucose in the absence (top) and presence (bottom) of BMP8B. In the absence of BMP8B the voltage response increased in 0.1 mM glucose; however, BMP8B blocked this response. C. Bar graph of the percentage change in resistance as glucose was lowered from 2.5 to 0.1 mM glucose in the absence (con) and presence (BMP) of BMP8B. BMP8B significantly decreased the percentage change in resistance. Paired t-test. *p = 0.01. White square: AdGI neurons; black dot: sustained (s) GI neurons. D. Bar graph of the percentage of nNOS expressing cells that also express the BMP receptor BMPR1a in males and females. E. Immunohistochemical images of the VMH from males (left) and females (right) showing colocalization of neuronal nitric oxide synthase (nNOS, green) and BMPR1a (red). Dapi nuclear stain in blue. White arrows indicate cells expressing both nNOS and BMPR1a. F. Hypothetical model for the effect of BMP8B on VMH GI neurons. Glucose inhibits VMH nNOS-GI neurons by inhibiting AMPK. BMP8B also exerts it's metabolic effects by inhibiting VMH AMPK. Thus, BMP8B may regulate blood glucose in females due to increased expression of BMPR1a on nNOS expressing VMH GI neurons compared to males. * p<0.01, independent students t-test.

## Description

The ventromedial hypothalamus (VMH) plays a key role in glucose and energy homeostasis (King, 2006). The VMH possesses specialized glucose sensing neurons whose activity is increased (glucose-excited; GE) or decreased (glucose-inhibited; GI) as extracellular glucose levels rise (Song et al., 2001). We have extensively characterized the role of VMH GI neurons in the sympathoadrenal counterregulatory response to insulin-induced hypoglycemia (Fioramonti et al., 2013; Fioramonti et al., 2010; Santiago et al., 2016; Song and Routh, 2006; Zhou and Routh, 2018). As glucose levels decline, the activity of the cellular fuel sensor AMP activated protein kinase (AMPK) increases in GI neurons leading to phosphorylation of neuronal nitric oxide synthase (nNOS) (Murphy et al., 2009). VMH activation of both AMPK and nNOS are required to restore euglycemia following insulin-induced hypoglycemia (Fioramonti, 2010; McCrimmon et al., 2008).

In addition to maintaining euglycemia, the VMH regulates energy expenditure through the sympathetic activation of brown adipose tissue (BAT) thermogenesis and white adipose tissue (WAT) browning (Xu et al., 2011). Bone morphogenetic protein (BMP) 8B, a member of the transforming growth factor (TGF) α-BMP superfamily, promotes BAT thermogenesis and WAT browning in females, but not males, by inhibiting VMH AMPK (Martins et al., 2016; Whittle et al., 2012). This effect may be mediated by the BMP receptor type 1A (BMPR1A). BMPR1A is linked to the SMAD signaling pathway which inhibits the liver kinase B1-AMPK pathway (Yadav et al., 2017).

We found that estrogen blunts activation of VMH GI neurons by inhibiting AMPK (Santiago et al., 2016). Thus, we hypothesize that, like estrogen, BMP8B via the BMPR1A potentiates glucose inhibition of VMH nNOS-GI neurons leading to reduced activation in low glucose. We further hypothesize that this pathway exhibits sexual dimorphism. To test this hypothesis, we determined whether application of BMP8B to brain slices from female mice containing the VMH blunted activation of VMH GI neurons in low glucose using whole cell patch clamp recording. Next, we determined if the BMPR1A was co-expressed by VMH nNOS neurons and if expression differed between the sexes.

Our data show that membrane potential depolarization of VMH GI neurons in low glucose was blocked by BMP8B ( Figure 1A ). VMH GI neurons are characterized by an increase in membrane resistance in low glucose that is indicative of increased excitability. BMP8B blocked this effect ( Figure 1B, C). There are 2 subtypes of VMH GI neurons: those that show sustained activation in low glucose (sustained or sGI) and those whose excitability returns to baseline in the continued presence of low glucose (adapting or AdGI) (Santiago et al., 2016). Figure 1 is an example of a VMH AdGI neuron; however, a similar effect was observed for both sGI and AdGI neurons ( Figure 1C ). Our data further show that over 60% of VMH nNOS expressing neurons in female mice co-express BMPR1A. Co-expression of nNOS and BMPR1A is significantly lower in male mice ( Figure 1D, E). These findings support our hypothesis that BMP8B activation of the BMPR1A on VMH nNOS-GI neurons reduces their activation in low glucose ( Figure 1F ). Further, that VMH nNOS-GI neurons may mediate some of the metabolic effects of BMP8B. Our observation that females show greater VMH colocalization of nNOS and BMPR1A is consistent with the literature showing a lack of effect of VMH BMP8B in males (Martins et al., 2016; Whittle et al., 2012). It is possible that expression is not sufficient in males to lead to physiological effects.

The role of BMP8B inhibition of VMH nNOS-GI neurons in whole body physiology requires further study. Our previous data link VMH GI neurons to increased blood glucose during stress (e.g., hypoglycemia or fasting) (Fioramonti et al., 2010; Murphy et al., 2009; Santiago et al., 2016; Song and Routh, 2006; Zhou and Routh, 2018). On the other hand, VMH BMP8B is associated with increased energy expenditure resulting from stimulation of BAT thermogenesis and WAT browning. In these studies glucose homeostasis was not evaluated (Martins et al., 2016; Rial-Pensado E et al., 2022; Whittle et al., 2012). Interestingly, our preliminary data suggest that silencing VMH nNOS increases body weight and blood glucose in females in association with decreased physical activity. However, BAT thermogenesis and WAT browning were unaffected (Hirschberg et al., 2024). It is possible that VMH nNOS-GI neurons represent only a subset of BMPR1A expressing neurons and that different neuronal populations control glucose homeostasis and BAT/WAT thermogenesis. Future studies are needed to define the relationship between BMP8B action on energy expenditure and glucose sensing in the VMH.

## Methods

Animals. Male and female 6 to 10-week-old C57BL/6J mice were housed on a 12:12 light-dark cycle with food and water provided ad libitum. All procedures were approved by the Rutgers Newark Institutional Animal Care and Use Committee (IACUC).

Electrophysiology.

Female 6 to 10-week-old C57BL/6J mice were anesthetized with sodium pentobarbital (60–80 mg/kg, i.p.) and transcardially perfused with ice-cold oxygenated (95%O2/5%CO2) N-methyl- d -glucamine (NMDG) perfusion solution (composition in mM: 110 NMDG, 2.5 KCl, 1.25 NaH 2 PO 4 , 30 NaHCO 3 , 20 N-2-hydroxyethylpiperazine-N′-2-ethanesulfonic acid (HEPES), 10 glucose, 2 thiourea, 0.5 CaCl 2 , 10 MgSO 4 ·7H 2 O, 5 Na-ascorbate, 3 Na-pyruvate (pH 7.3–7.4, osmolarity adjusted to 310–315 mOsm). Brains were rapidly removed, placed in ice-cold (slushy) oxygenated NMDG perfusion solution and 300 μm coronal slices containing VMH neurons were made on a vibratome (7000 smz2, Vibroslice, Camden Instruments, Camden, UK) as previously described (Teegala et al., 2023). The brain slices were then transferred to a pre-warmed (34 °C) initial recovery chamber filled with 150 ml of NMDG perfusion solution. After transferring the slices, Na + was reintroduced following the Na + -spike method (Ting et al., 2018). Slices were then transferred to HEPES-artificial cerebrospinal fluid (aCSF) holding solution (composition in mM: 92 NaCl, 2.5 KCl, 1.25 NaH 2 PO 4 , 30 NaHCO 3 , 20 HEPES, 2.5 glucose, 2 thiourea, 5 Na-ascorbate, 3 Na-pyruvate, 2 CaCl 2 ·2H 2 O, and 2 MgSO 4 ·7H 2 O (pH 7.3–7.4, 310–315 mOsm) and allowed to recover for 1 h at room temperature prior to whole-cell current recording using the recording aCSF (composition in mM: 124 NaCl, 2.5 KCl, 1.25 NaH 2 PO 4 , 24 NaHCO 3 , 2.5 glucose, 5 HEPES, 2 CaCl 2 ·2H 2 O, and 2 MgSO 4 ·7H 2 O (pH 7.3–7.4, 310–315 mOsm).

Data were collected in the current clamp mode using using pClamp v11.3 software and an AxoPatch 200B amplifier and digitized with a DigiData 1500B AD converter (Molecular Devices). Neurons were visualized using infrared differential interference contrast microscopy. aCSF was perfused at a rate of 3-5mL/minute. Borosilicate pipettes were pulled to 3-5mOhm resistance and back filled with pipette solution (K+-Gluconate 128mM, EGTA 0.5mM, KCl 10mM, HEPES 10mM, MgCl2. 6H20 4mM, CaCl2 2H20 0.5mM, Na2ATP, and 0.4 Na2GTP 2 mM, osmolarity 290-300 mOsm, pH 7.1-7.2). A 300msec -10 or -20pA hyperpolarizing current was injected every 3 seconds. GI neurons were identified by a reversible depolarization and increase in cellular resistance as glucose decreases from 2.5 to 0.1 mM. The voltage change to the injected current was calculated for 10 consecutive voltage responses at the height of the response to low glucose. For sGI, these ten responses were taken from the very end of the 10-minute recording. For AdGI neurons the 10 responses at the height of the neuron response were averaged. Ohm's law (V=IR) is used to calculate the resistance based on the value of injected current in Amps (I) and the average voltage response in Volts (V). The change in cell resistance was required to be at least 15% in response to low glucose to characterize the neuron as GI.

IHC:

30µM sections: Male and female mice were perfused with 4% paraformaldehyde (PFA). Brains were kept for 24hr in 4% PFA, cryoprotected with 15 and 30% sucrose solution in 0.01M phosphate buffered saline (PBS) and stored in -80°C in OCT (tissue tek, Sakura). After sectioning 30µM sections, the tissue was blocked with 10% BSA in PBS containing 0.01% Triton –X-100 for one hour. Post blocking, the slices were probed with primary antibody. Tissue was washed 3 times for 10 min in PBS containing 0.05% Tween 20 before placing in secondary antibody. Slices were mounted on slides with mounting media containing nuclear counterstain (ProLong™ Gold Antifade Mountant with DNA Stain DAPI, Thermo Fisher P36931) and cover glass.

Materials: Bone Morphogenic Protein 8B (BMP8B) human (Millipore Sigma, SRP 6322, Burlington, MA), Antibodies: primary rabbit anti-NOS1 (1:200, Cell Signaling Technology C7D7,Danvers, MA), secondary Alexa Fluor™ 488 (1:500, Donkey anti-Rabbit; A21206, Invitrogen, Waltham, MA); primary mouse Anti-BMPR1A (1:250, Santa Cruz Biotechnology, sc293175, Dallas, TX), secondary Alexa Fluor™ 594 (1:500, Donkey anti -Mouse; A21203, Invitrogen, Waltham, MA)

Cell Counting:

Images were obtained using the Nikon A1R Confocal Laser Microscope System. BMPR1a-, nNOS-positive cells and cells that had co-localization of both BMPR1a and nNOS were counted manually using the 3D object counter in ImageJ.

Statistical analysis

All statistical analyses were performed using GraphPad Prism v8. Comparisons between groups were made using an independent t-test and within groups using a paired t-test. The values were expressed as the means ± SEM. The differences with p<0.05 were considered statistically significant.

## Reagents

STRAIN SPECIES Available from

C57BL/6J mouse Jackson Laboratories

ANTIBODY Animal/Clonality Description

NOS1 rabbit Anti-NOS1 primary

BMPR1A mouse Anti-BMPR1A primary

Alexa Fluor™ 488 Donkey anti-rabbit secondary for NOS1

Alexa Fluor™ 594

Donkey anti-mouse secondary for BMPR1A

CHEMICALS CATALOG# Available from

BMP8B (human) SRP 6322 Millipore Sigma
